# In vivo and ex vivo range of motion in the fire salamander *Salamandra salamandra*

**DOI:** 10.1111/joa.13738

**Published:** 2022-08-20

**Authors:** Eva C. Herbst, Enrico A. Eberhard, Christopher T. Richards, John R. Hutchinson

**Affiliations:** ^1^ Palaeontological Institute and Museum University of Zurich Zurich Switzerland; ^2^ Structure and Motion Laboratory Royal Veterinary College London UK

**Keywords:** gait, joint mobility, motion capture, range of motion, rotoscoping, salamander

## Abstract

Joint range of motion (RoM) analyses are fundamental to our understanding of how an animal moves throughout its ecosystem. Recent technological advances allow for more detailed quantification of this RoM (e.g. including interaction of degrees of freedom) both in ex vivo joints and in vivo experiments. Both types of data have been used to draw comparisons with fossils to reconstruct locomotion. Salamanders are often used as analogues for early tetrapod locomotion; testing such hypotheses requires an in‐depth analysis of salamander joint RoM. Here, we provide a detailed dataset of the ex vivo ligamentous rotational joint RoM in the hindlimb of the fire salamander *Salamandra salamandra*, using a new method for collecting and visualising joint RoM. We also characterise in vivo joint RoM used during walking, via scientific rotoscoping and compare the in vivo and ex vivo data. In summary, we provide (1) a new method for joint RoM data experiments and (2) a detailed analysis of both in vivo and ex vivo data of salamander hindlimbs, which can be used for comparative studies.

## Graphical abstract

Here, we captured a detailed dataset of hip and knee joint range of motion (RoM) in *Salamandra salamandra*. The correspondence between in vivo (walking) and ex vivo (ligamentous) joint RoM varied with joint and rotational axis, with knee flexion/extension being most similar between in vivo and ex vivo specimens. This dataset can be used as reference for studies investigating joint mobility and gait in extinct animals.

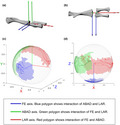

## 1 INTRODUCTION

Many aspects of an organism's biology, including osteology, soft tissue and behaviour, interact to produce movement in animals. One method to analyse locomotion in animals is to quantify joint range of motion (RoM). Investigating the relationship between ex vivo and in vivo RoM is important; measuring joint RoM ex vivo allows researchers to quantify the effects on RoM of different soft tissues or bony morphology, whereas in vivo RoM allows quantification of the poses used to facilitate a specific gait. Previous studies have shown that animals typically use a subset of the possible (ex vivo) joint RoM during different locomotor behaviours (Arnold et al., 2014; Kambic et al., 2017; Manafzadeh et al., 2021; Ren et al., 2008). Furthermore, the ex vivo joint RoM differs based on how much soft tissue is kept intact (Arnold et al., 2014; Hutson & Hutson, 2012, 2013).

Here, we characterise the ex vivo and in vivo joint RoM in the hip and knee of Fire Salamanders *Salamandra salamandra* Linnaeus 1758. For the ex vivo experiments, we tested joint RoM in cadavers with only the ligaments and joint capsule kept intact, and for the in vivo experiments, we tested the joint RoM used during walking. We chose to use ligament‐only data because it encapsulates the 3D bones‐only RoM in various specimens including *S. salamandra* (Pierce et al., 2012). For fossil comparison, bones‐only joint RoM is commonly investigated because soft tissues are rarely preserved (Manafzadeh & Padian, 2018; Pierce et al., 2012). However, bones‐only RoM often does not provide sufficient constraints on hip long axis rotation, for example in Nile crocodile RoM comparisons (Pierce et al. (2012) Supplementary Info). Therefore, we measured ligamentous RoM in the salamanders. The ex vivo dataset was collected using a custom rig, and joint RoM was visualised using the new spherical frame projection (SFP) method, which incorporates interaction of degrees of freedom (the effect of the rotations about each axis on the rotational ranges of the other axes) and provides an accurate representation of the real distances between poses (see Herbst, Eberhard, et al., 2022).

The goals of this study were threefold. First, it is a case study using the methodology from Herbst, Eberhard, et al. (2022). Second, we wanted to determine how in vivo hindlimb RoM during walking compares to ex vivo ligamentous joint RoM. We predict that, as in various other animals such as elephants, iguanas and guineafowl (Arnold et al., 2014; Kambic et al., 2017; Ren et al., 2008), the in vivo RoM in salamanders will be a subset of the ex vivo RoM, especially because we removed several soft tissue structures (skin and muscles) for the ex vivo experiments. We also aimed to investigate whether the general in vivo RoM is “centred” in the ex vivo range. A recent study (Manafzadeh et al., 2021) demonstrated that in guineafowl and alligator hip and knee joints, the in vivo and ex vivo joint RoM shared certain patterns: the cosine‐corrected in vivo flexion/extension (FE) range was centred within the middle 50%–75% of the ex vivo FE range (depending on taxon). Additionally, the in vivo ranges fell on the adducted side of ex vivo hip RoM and the adducted side of in vivo knee and ankle RoM. Manafzadeh et al. (2021) proposed that these patterns could be used to reconstruct locomotion in extinct archosaurs. We therefore wanted to determine whether similar patterns also hold true for salamanders.

The third aim of our study was to provide a detailed reference dataset (including interaction of degrees of freedom) for future studies comparing fossil RoM to Fire Salamander RoM. Salamanders have often been used as possible analogues to early tetrapods, due to morphological similarities (Ashley‐Ross, 1994; Kawano & Blob, 2013; Schaeffer, 1941). Osteological range of joint motion is valuable in gaining insight into the locomotor capacities of extinct animals but is most valuable when put into the context of other constraints on RoM. Although we do not know the extent to which soft tissue may have restricted joint RoM in a fossilised taxon, and we cannot measure behaviour, we can nonetheless test different joint orientations (poses) and thereby exclude locomotor behaviours that were not possible (based on bone disarticulation and interpenetration). To exclude non‐viable locomotor behaviours in fossil reconstructions, data from extant animals performing such behaviours are needed for comparison. The results of our study on salamander hind limb joint RoM can be used to determine what joint poses are used in a typical salamander‐like walk. In Herbst, Manafzadeh, and Hutchinson (2022), we use this data to investigate the possibility of salamander‐like hindlimb configurations in the early tetrapod *Eryops*.

## 2 MATERIALS AND METHODS

This section is organised as follows: First, we give an overview of the SFP for visualising joint RoM. Next, we discuss the specimens. Then, we discuss the ex vivo experiments, anatomical coordinate systems, and in vivo data collection via rotoscoping.

### 2.1 Spherical frame projections to visualise joint range of motion

We represent the joint RoM using SFPs. SFPs are new visualisations to illustrate the entire rotational pose space, including interaction of degrees of freedom, for a joint (Herbst, Eberhard, et al., 2022). Essentially, the rotation of the ACS of the distal bone(s) of the joint is traced on a sphere, relative to the ACS of the proximal bone of the joint (which remained stationary in the experiments; Movie S1). SFPs avoid the gimbal lock difficulties of Euler angles and as visual representations, they are useful because the distances between two poses (i.e. between axis tips) in the SFP are more representative of real differences between the poses than Euler–Euler–Euler angle plots (Herbst, Eberhard, et al., 2022, but see Manafzadeh and Gatesy (2020) for a method of how to correct for this distortion in Euler angle plots).

### 2.2 Specimens

The Fire Salamander specimens were purchased from an animal supplier (Ameyzoo) and were euthanised and frozen (−20°C) after originally being used for electromyography, force plate and X‐ray video experiments (details in Pierce et al., 2020). Specimen numbers in this text are the same numbers used in Pierce et al. (2020). Two of these specimens (salamander 06 and salamander 08) were used in both studies (salamander 12 was also used in the Pierce et al. study but only for the forelimb).

### 2.3 Ex vivo experiments

The ex vivo RoM data were measured using a custom‐built motion capture rig (Herbst, Eberhard, et al., 2022). We dissected individual joints and then secured the bones at either end of the joint to the rig. One end of the rig was stationary, containing a load cell and the other one was moveable, containing reflective markers so that we could track the joint movement with the motion capture system. The positions of the markers were then transformed to a distal anatomical coordinate system (ACS) to obtain joint rotations relative to the proximal ACS: flexion/extension, abduction/adduction and long axis rotation (from here on abbreviated as FE, ABAD and LAR). The data collection procedure is described in more detail below.

**FIGURE 1:**
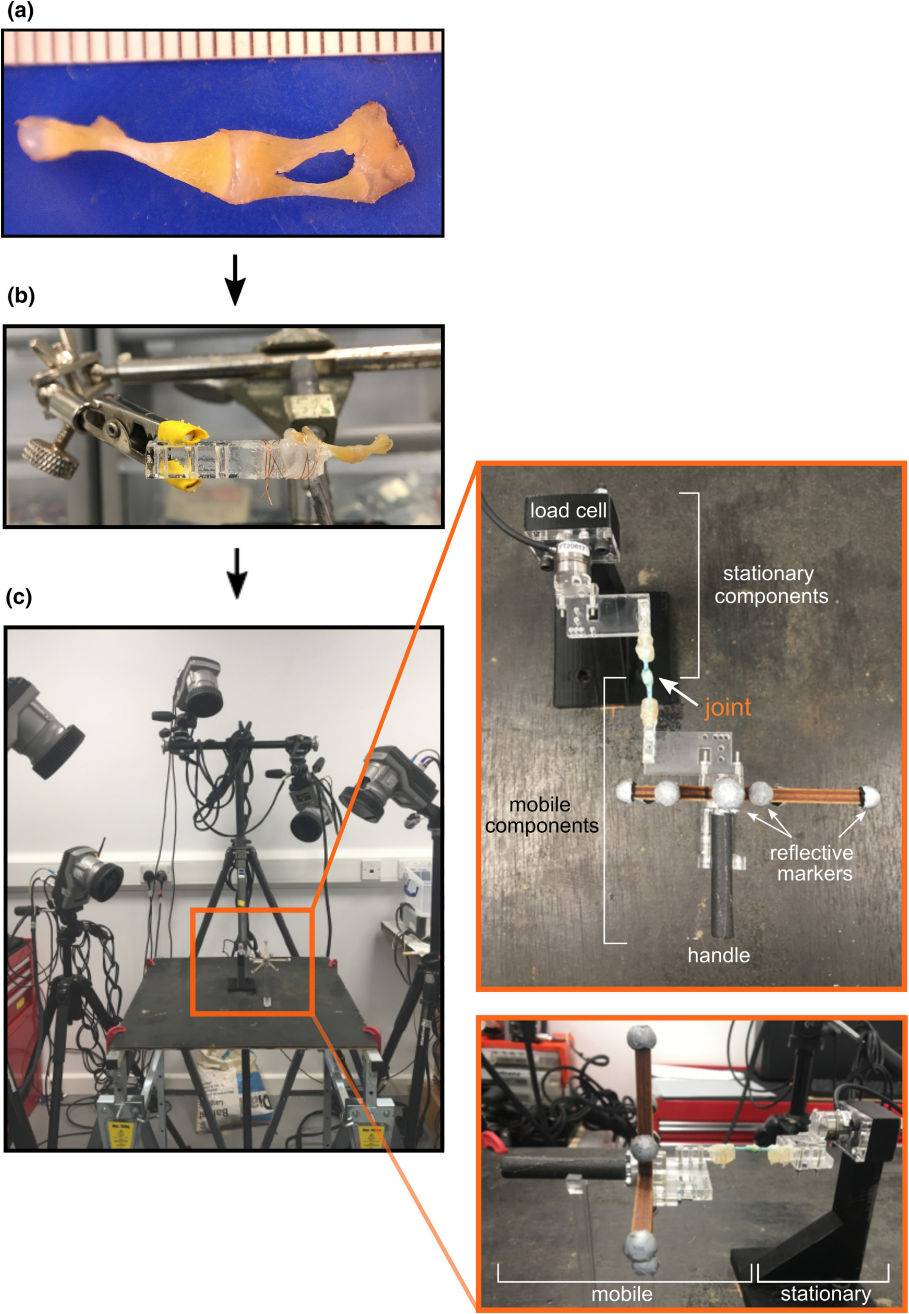
Rig setup for ex vivo experiments. (a) Muscle and skin were removed; (b) joint was rigged to plates; (c) plates were screwed into rig. The proximal bone was attached to the load cell side of the rig (stationary), and the distal bone(s) was attached to seven motion capture markers and a handle to move the joint. The knee joint in (c) was from a trial with a frog but shows the same setup used for the salamander experiments.

For the ex vivo joint RoM, we dissected five knee joints and five hip joints of *S. salamandra*, removing the skin and most muscles and keeping the ligaments and joint capsule intact (Figure S1). To rig the joint for data collection, we attached the proximal and distal bones to acrylic plates with glue and wire. Details on dissections and rigging are given in the Appendix. We then attached the plates to the rig with two screws; the proximal plate was attached to the stationary part of the rig and the distal section was attached to a moveable handle with reflective motion capture markers (Figure 1; see Herbst, Eberhard, et al., 2022 for more details on the joint RoM rig).

For the knee joint, the tibia and fibula were treated as a unit, and glued together to a single plate. Due to the difficulty of measuring independent movements of the tibia and fibula in such small specimens, we took care to maintain the relative positions of these bones such that they could be treated as a singe rigid body (see Figure S6b,d, showing similar relative positions of these bones between specimens). This simplification enabled us to focus on measuring how the distal bone(s) at a joint moved relative to the proximal bone. Knee joints were rigged so that the null (zero, or “reference”) pose was an extended joint. In the null pose, a flexion motion at the knee joint (relative to the null pose) brought the distal tibia up towards the ceiling (Movie S1). The hip joints were rigged so that the acetabulum was facing upwards away from the table in the null pose. The anatomical coordinate systems used for the bones are described in more detail below.

We recorded the joint RoM using a Qualisys motion capture system with six Opus cameras (Qualisys AB) and custom rig. A live feedback system of covered poses and associated torques enabled us to ensure covering of the whole pose space and avoiding joint damage (Herbst, Eberhard, et al., 2022).

Other studies often quantify RoM as the maximal values about these three axes, but biological motion is not planar. The rotation of a joint about one axis can influence the RoM about another axis (Haering et al., 2014; Kambic et al., 2017; Manafzadeh & Padian, 2018). Therefore, we moved the joint through three‐dimensional space, using combinations of rotations to capture interaction of degrees of freedom. With three anatomical axes, there are six possible sequences: FAL, FLA, AFL, ALF, LFA, LAF. The sequence itself represents the hierarchical order in which angles were manipulated. For example, a sequence of FAL would first sweep the range around the FE axis, keeping the ABAD and LAR axes constant. Then, the ABAD axis would be rotated incrementally, and the FE motions repeated at each ABAD position. Finally, the LAR was adjusted incrementally, and the sweeps of FE and ABAD repeated, until rotation limits around LAR were reached. In the example sequence FAL, the FE limit was approached with the highest frequency, ABAD with moderate frequency and LAR the least frequently. Therefore, the FAL sequence was biased in primarily straining tissue structures around the FE axis. We therefore randomised the order of the sequences between trials to balance the bias and minimise tissue strain.

For each specimen, we conducted nine trials (three for planar movement for reference and all subsequent trials quantifying interaction of degrees of freedom) or until the joint dislocated, the joint capsule opened or ligaments visibly loosened. Usually, such damage occurred before trial nine, because the salamander joints were small and fragile and therefore the joint capsule and ligaments were under repetitive stress (especially because the aim was to sample all possible poses, not just check maxima of planar motion). Consistent with prior work, on occurrence of any of visible damage, ligament tearing noise, sudden decrease in resistance, sudden increase in rotational RoM, or noticeable increase in translation occurred at the joint, we stopped recording and discarded the data for that trial (Hutson & Hutson, 2012; Manafzadeh, 2020). Note that we did not analyse translational RoM and only focused on rotational RoM. Sudden increases in RoM were also visible in the live feedback spheres (see above): if suddenly poses “jumped” outside the previous pose space without any gradual continuity, this indicated some sort of damage. The trials that included loosening or damage were not included in the final analysis. Sufficient trials were defined as the three trials about the three anatomical axes and at least two trials including interaction of degrees of freedom. Some specimens did not have sufficient trials and therefore got excluded. The four specimens with sufficient trials for the hip in this analysis were salamander 10, salamander 12, salamander 13 and salamander 14. The two specimens used for the knee analysis were salamander 06 and salamander 08. All joints were from the right side except one hip, whose data were transformed to create a right side SFP and Euler angles matching the right side.

A custom Matlab script captured the orientation of the marker tree from Qualisys Track Manager (QTM) in rotation matrix format at 30 Hz, and six‐channel force sensor data from an ATI Nano17 6‐axis force and torque (F/T) transducer at 1.5 kHz.

After all trials were completed, we unscrewed the acrylic plates (with bones) from the rig and disarticulated the joint. We used a microCT scanner (Bruker Skyscan 1172; 13.46 μm resolution with a source voltage of 49 kV and source current of 141 μA) at the Royal Veterinary College to scan the bones and plates.

### 2.4 Anatomical coordinate systems and data transformations

The motion capture data needed to be transformed from the marker positions in world space to an anatomical coordinate system (ACS). An ACS is a set of three axes defined relative to the morphology of the bone so that movement about the distal anatomical coordinate system relative to the proximal one characterises the anatomical motions of FE, ABAD and LAR (Gatesy et al., 2022; Grood & Suntay, 1983; Kambic et al., 2014). To quantify the orientation and position of the bones in the trials, we used Rhino (v6, Robert McNeel and Associates) to assign ACSs to the bones and acrylic plates.

We ensured our ACSs were compatible with the conventions used in previous rotoscoping studies. Maya (the programme we used for rotoscoping, Autodesk, San Rafael, CA, USA) calculates rotations in a *Z*‐*Y*‐*X* rotation order; the *Z*‐axis is usually defined as the axis in which the most motion is expected (Brainerd et al., 2010). Previous studies of salamander motion have found that femur retraction/protraction (here referred to as “flexion/extension” or “FE”) and knee FE had a wide range of movement over the stride cycle; e.g. (Karakasiliotis et al., 2013). Therefore, we created right‐handed ACSs where Z = FE, Y = ABAD and X = LAR, consistent with several other XROMM studies (Gatesy et al., 2022; Kambic et al., 2014, 2017; Manafzadeh & Padian, 2018).

ACSs are usually based on geometric primitives fit to the bones (Bishop et al., 2021; Gatesy et al., 2022; Kambic et al., 2014, 2017; Manafzadeh & Padian, 2018). However, we deemed this method unsuitable for most of the salamander bones, because salamanders have large cartilage caps and the osseous ends of most bones are flat; geometric primitives are not good approximations for the bony morphology (except for the acetabulum). The specimens were not contrast‐stained, and segmentation of the cartilage proved unfeasible. Therefore, we developed a new method in Matlab to define ACSs based on points placed on the bone in Rhino (details below). Our sensitivity studies of this method showed low inter‐user bias and high agreement with other methods (see Appendix). For all scans, we also placed points to assign a coordinate system for the mounting plate, to determine the orientation of the bone relative to the plate for data transformations.

#### 2.4.1 Hip anatomical coordinate systems

##### Acetabular anatomical coordinate systems

We used geometric primitives to help place the pelvic and acetabular anatomical coordinate systems (ACSs), because of the roughly spherical acetabula and a roughly cylindrical vertebral body of the sacral vertebra. Our cadaver scans of the pelvis only contained one side of the hip girdle, because we dissected the other side and sacral vertebra off for the sake of stability in mounting the specimens to the rig. Therefore, we used the full body scan of the rotoscoped individual (specimen 22, see “*In Vivo* Joint RoM”) to assign the hip ACS, and then transferred that hip ACS to the cadaver specimens using CloudCompare software (CloudCompare version 2.5.4.1, GPL software, retrieved from http://www.cloudcompare.org/).

To create the pelvis and hip ACSs, we first fit a cylinder to the vertebral body of the sacral vertebra to calculate the anteroposterior axis (*X‐*axis) for the pelvis. We wanted to use the pitch (relative to the pelvis) to define the pelvis *X*‐axis. However, this vertebra had both pitch and yaw (due to flexibility between the sacral vertebra and pelvis). Disregarding the yaw, we used the pitch to define the pelvis *X*‐axis to obtain an anteroposterior axis through the midline of the pelvis. To get a symmetric *X*‐axis for the pelvic ACS, we used Rhino to calculate a line with the pitch of the sacral vertebra cylinder but perpendicular to the mediolateral axis.

**FIGURE 2:**
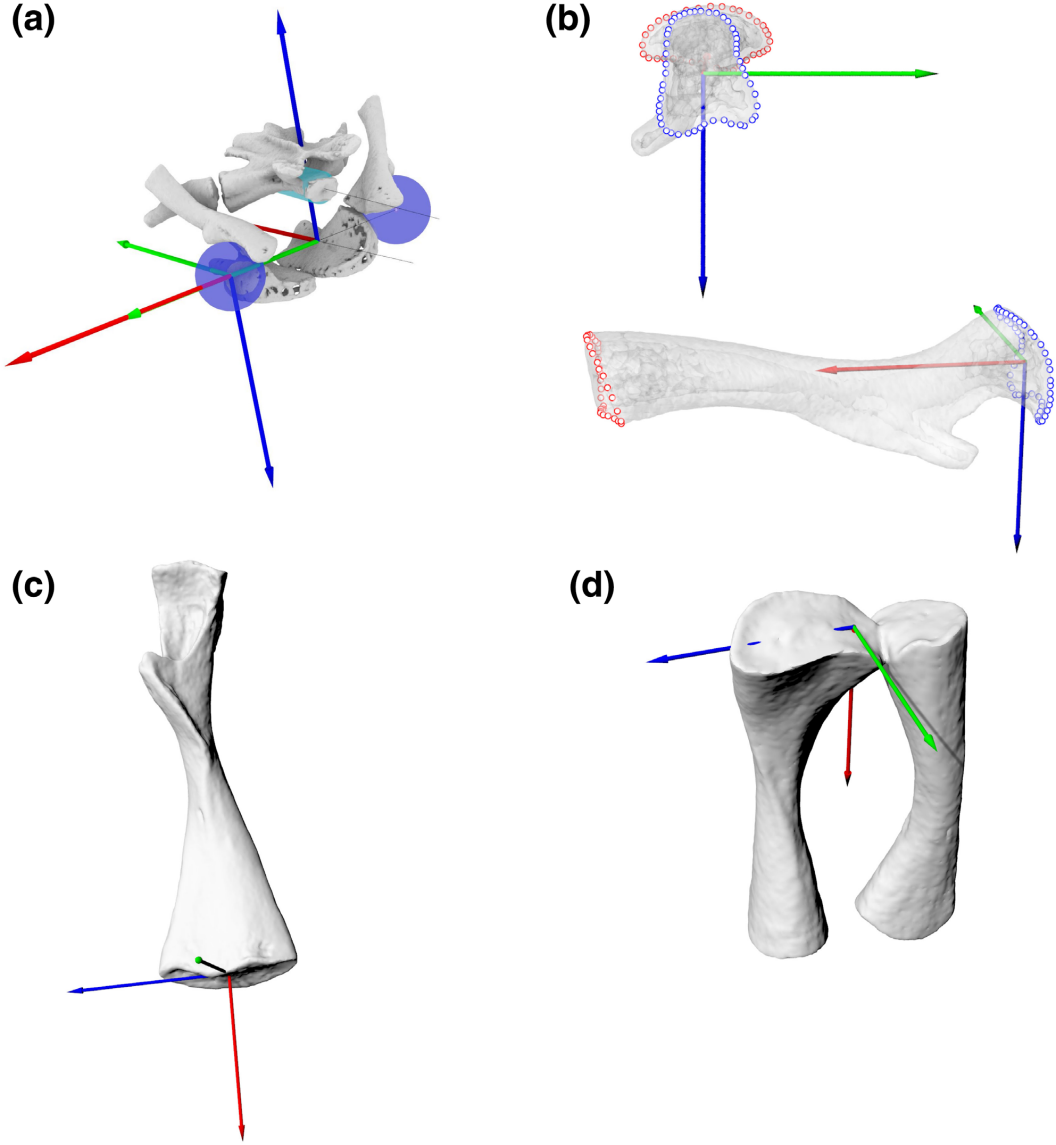
(a) Pelvic and acetabular ACSs, created via fitting a sphere to the acetabular surfaces and a cylinder to the sacral vertebra. The anteroposterior axis was based on the pitch of the cylinder, the mediolateral axis was the line connecting the centroids of the acetabular spheres, and the dorsoventral axis was orthogonal to the mediolateral and anteroposterior axes. (b) Proximal femur ACS, (c) distal femur ACS, (d) proximal tibia/fibula ACS, created by fitting lines to points placed on the perimeters of the proximal and distal bone surfaces. Blue = FE, green = ABAD, red = LAR.

Then, we fitted spheres to both hip acetabula and calculated the centroids for each (Figure 2a). The geometric shape fitting involved segmenting out the articular surface in Meshlab (Meshlab v1.3.3, Cignoni et al., 2008), and then using Matlab code (Bishop et al., 2021) to fit the desired shape to the mesh of the articular surface. The centroid for each acetabular sphere defined the origin of the ACS for that hip joint, and the point midway between these centroids was the origin of the pelvis ACS. A line was fit through the left and right centroids, to act as the mediolateral axis (*Y*) of the pelvis. The dorsoventral axis (*Z*) of the pelvis was orthogonal to the mediolateral and anteroposterior axes. The pelvis ACS axes definitions and directions followed the pelvis ACS established by Kambic et al. (2014); also see Gatesy et al. (2022). For the pelvis ACS, the *X*‐axis pointed caudally; positive rotation about this axis resulted in roll to the left (Figure 2a). The *Y*‐axis pointed right; positive rotation about this axis was upward pitch. The *Z*‐axis pointed up; positive rotation about this axis was yaw to the left. The acetabular ACSs were based on the pelvis ACSs, but translated to the acetabular origins and rotated to capture the correct anatomical motions.

##### Proximal femur ACS

We used the programme Rhino and custom Matlab code to place points on the perimeter of the proximal and distal ends of the bone and to fit lines through these points (Figure 2b). For each bone, the mean of the points on the proximal and distal surfaces was calculated. The *X*‐axis (LAR) was the line from the mean of the proximal points to the mean of the distal points. The temporary *Z*‐axis was a line fit through the proximal points. The *Y*‐axis was the cross product of the *X*‐axis and the *Z*‐axis. Because of the morphology of bone, one of the axes needed to be recalculated to produce orthogonal axes. We recalculated the *Z*‐axis (FE) as the cross product of the *X* and *Y* axes to ensure that all of the axes were orthogonal, and to keep the *X*‐axis (LAR) aligned with the anatomical long axis of the bone; similar to Kambic et al. (2014). Directions were adjusted using a reference point on the ventral side of the femur so that in a null pose with the femur extended laterally from the hip, the femoral axes' orientations were the same as those of the acetabulum.

#### 2.4.2 Knee ACSs

##### Distal femur and proximal tibia/fibula ACS

A single ACS was created for the proximal tibia and fibula because, in the ex vivo and rotoscoping experiments, the tibia and fibula were treated as a unit (a single rigid body—“crus”), and their motion relative to the femur was analysed. For each bone (the femur and the tibia/fibula unit) the mean of the points on the proximal and distal surfaces was calculated. The mean of the distal points formed the origin of the ACS for the proximal bone at the joint (Figure 2c), and the mean of the proximal points formed the origin of the ACS for the distal bones (tibia/fibula, Figure 2d).

For the femur, a line was fit along the points at the distal surface. For the distal bones of the joint (tibia and fibula for the knee joint), a line was fit along the points of the proximal surface. These lines formed the preliminary *Z* (FE) axes of the ACSs of the bones. This point placement and line calculation worked here because in the salamander knee, the distal femur is roughly an ellipsoid with the longer dimension along the FE axis, and the proximal tibia and fibula together also have the longest dimension along this axis. For each bone, a line was also drawn from the mean of the proximal points to the mean of the distal points, forming the preliminary *X*‐axis (LAR). The *Y*‐axis (ABAD) was defined as the cross product of the *X*‐axis and the *Z*‐axis.

However, as in the proximal femur, the preliminary *Z* and *X* axes were not exactly 90° relative to each other (because they were calculated based on the morphology of the bones). For the distal femur, we kept the *Z*‐axis (FE) as the line fit through the distal points, so that this axis was aligned along the longest dimension of the distal surface of the femur, reflecting the distal femur's morphology. To make the axes orthogonal, we recalculated the *X*‐axis as the cross product of the *Y* and *Z* axes. For the tibia/fibula, we kept the *X*‐axis (LAR) aligned to the morphology (i.e., from the mean of the proximal points to the mean of the distal points). We recalculated the *Z*‐axis (FE) as the cross product of the *X* and *Y* axes because, for the proximal tibia/fibula ACS, the *X*‐axis (long axis) was more anatomically intuitive to define, and we wanted to keep this axis calculation along the anatomical long axis. If instead the *X*‐axis were recalculated, the long axis would be skewed towards the tibia. The final axes are shown in Figure 2c (distal femur) and 2D (proximal tibia/fibula). Defining the *Z*‐axis of the distal femur ACS and the *X*‐axis of the proximal tibia ACS based on the morphology is consistent with Kambic et al. (2014), which established ACS calculation conventions broadly used in subsequent studies (Gatesy et al., 2022; Kambic et al., 2017; Manafzadeh & Padian, 2018).

In a null pose position with the knee extended, the directions of the axes for the distal femur ACS and proximal tibia/fibula ACS were defined as follows. For the right knee joint, the *Z*‐axis (FE) pointed cranially; positive rotation about the *Z*‐axis was flexion of the knee. The *Y*‐axis (ABAD) pointed away from the flexor surface of the knee joint; positive rotation about the *Y*‐axis was abduction of the knee. The *X*‐axis (LAR) pointed towards the distal end of the bone(s); positive rotation about the *X*‐axis was external rotation.

#### 2.4.3 Transforming the Qualisys data

The data from the motion capture markers needed to be transformed to the ACSs. We calculated the necessary transformations from motion capture datapoints to the acrylic plate from the dimensions of the rig, and then used CT scanning to determine the position and orientation of the bone relative to the acrylic plate. This enabled us to capture the motion of the distal bone relative to the proximal bone. The whole workflow from scanning to the final data is given in our companion paper (Herbst, Eberhard, et al., 2022).

### 2.5 In vivo joint RoM

In scientific rotoscoping, CT scanned bones of an animal are aligned to two biplanar X‐ray videos, to investigate three‐dimensional motion of the bones (Gatesy et al., 2010). Using this method, we quantified the observed in vivo hip and knee joint RoM of one individual (salamander 22) of *S. salamandra* during walking, with details as follows. ACSs were defined by the same equations and methods as for the ex vivo analysis.

#### 2.5.1 Rigging

There are several methods to rig and rotoscope an animal moving (Gatesy et al., 2010). We developed a method that allowed us to measure the motion of the ACS of the tibia/fibula relative to the distal femur's ACS. To do this, we first set up animation joints (in Maya 2019 software, Autodesk) for the pelvis, right hip and right knee. These formed a hierarchy, with the more distal joints parented to the more proximal joints. We used constraints to position these animation joints based on the ACSs. For animals with congruency between the bones at the joint (i.e., with less cartilage than salamanders), at the null pose, the ACSs of the proximal and distal bones are aligned (Kambic et al., 2014). Because of substantial amounts of cartilage at the joints of salamanders, we added some translations between the bones, based on the spacing from the full body CT scan of salamander 22. We did not use the rotations of these animation joints for analysis; instead, we measured the relative motions of the ACSs. However, making the animation joints as anatomically accurate as possible is important because it reduces the amount of translations required during the rotoscoping. We positioned the hip animation joint at the acetabular ACS, and oriented it to the proximal femur ACS. For the knee, we positioned the animation joint half‐way between the distal femur and proximal tibia/fibula ACS, because both the proximal and distal articular surfaces at the joint had a roughly similar amount of cartilage, so we expected the centre of rotation to be halfway between these ACSs. Bones were parented to the joints, and the ACSs point and orient constrained to the bones, so that both bones and ACSs followed along when the joints were rotated. We used the oRel command (XROMM_MayaTools, https://bitbucket.org/xromm/xromm_mayatools/wiki/Home) to measure the relative motion of the distal ACS relative to the proximal ACS of the joint so that we could compare the results to our ex vivo experiments, and generate a SFP to visualise the results of the rotoscoping analysis.

#### 2.5.2 Rotoscoping

**FIGURE 3:**
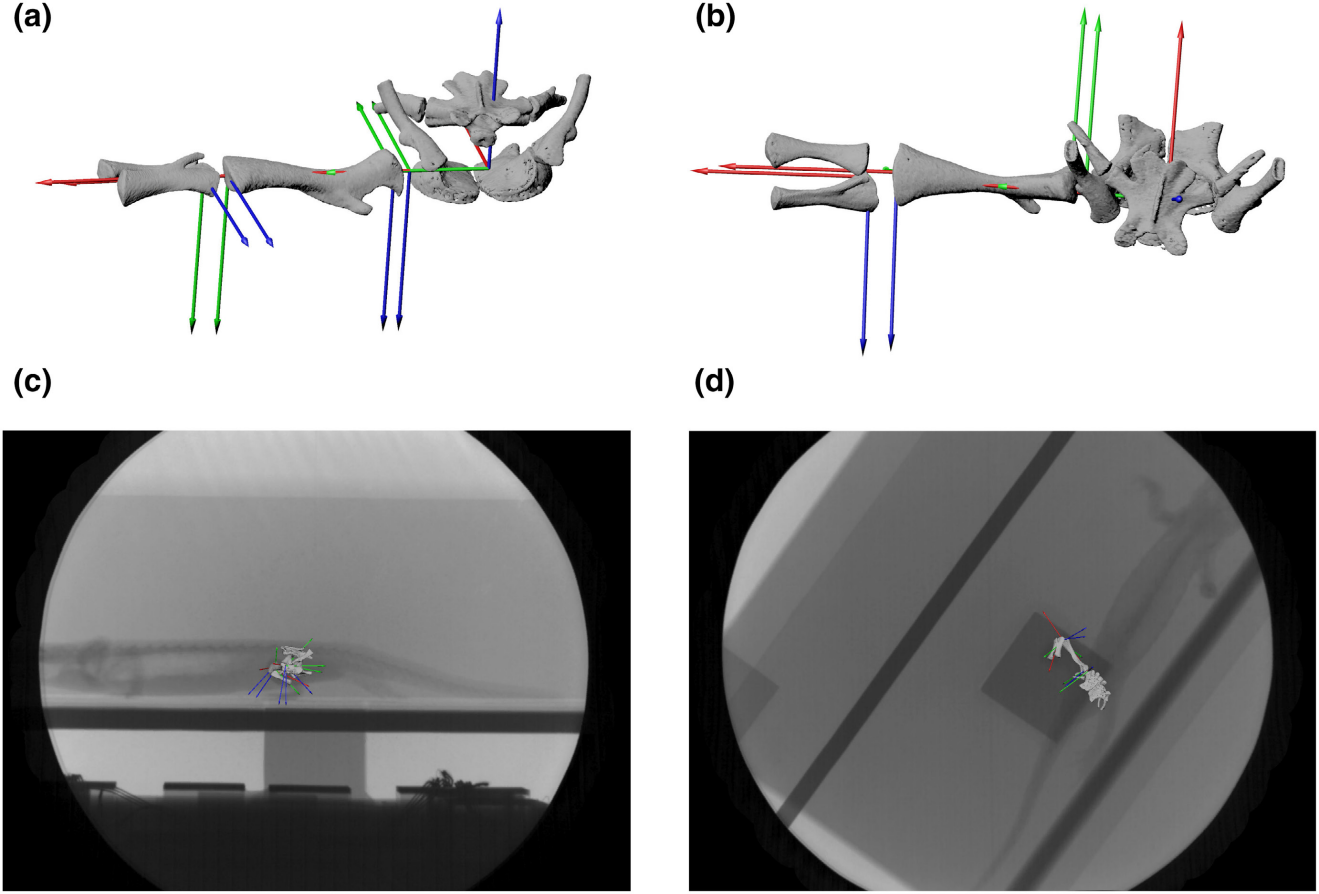
Rotoscoping setup. Null pose for salamander 22 in (a) anterior and (b) dorsal views. (c, d) Virtual cameras in Maya showing X‐ray videos in lateral (c) and ventral (d) views.

We rotoscoped one stride cycle (401 frames) of the right hindlimb of *S. salamandra* specimen 22 (Figure 3). The biplanar X‐ray videos were taken in 2014 in Jena, Germany by Jeffery Rankin, Stephanie Pierce and John Hutchinson for kinematic and force plate experiments (Pierce et al., 2020 and unpublished study). The salamander was moving over level ground at room temperature. Experiments were approved by the Royal Veterinary College's Animal Welfare and Ethics Review Board (AWERB‐A‐2013‐5064). See Pierce et al. (2020) for further information on animal care. The X‐ray videos were recorded at 500 Hz and 130 mA, the dorsoventral X‐ray was 40 kV and the mediolateral X‐ray was 50 kV. We converted the X‐ray video to TIFF images, enhanced contrast in ImageJ and used the XMALab procedure for undistortion and calibration (Knorlein et al., 2016, https://bitbucket.org/xromm/xmalab/wiki/Home). Where necessary, we added translations of the bones to align the bones to the X‐ray videos. However, we did not analyse these translations, as we are only investigating rotational RoM for this study (and did not exhaustively sample translations in the ex vivo dataset).

### 2.6 Data analysis

For the ex vivo data, we created the SFPs using the methods described in Herbst, Eberhard, et al. (2022). For the in vivo data, we first converted the Euler angles for each video frame from rotoscoping in Maya to a rotation matrix to visualise the rotoscoping output as an SFP.

To quantitatively analyse our SFPs, we also converted our ex vivo data to Euler angles, using Z‐Y‐X rotation order (FE calculated first). All data were rounded to the nearest degree. To include interaction of degrees of freedom in these results, we report the minimum and maximum values for each rotational axis, and the corresponding angles about the other two axes at that pose (Tables 1 and 2). Movie S1 shows an animation of a hypothetical knee ex vivo experiment, with the SFP generated as the joint is moved.

**TABLE 1:** Ex vivo and in vivo hip RoM. Euler angles for max and min values are listed in a *Z*, *Y*, *X* format, corresponding to FE, ABAD, LAR

	Max FE	Min FE	Range FE	Max ABAD	Min ABAD	Range ABAD	Max LAR	Min LAR	Range LAR
Sal10 ex vivo	**29**, 6, −73	−**85**, 24, −97	114	−26, **57**, −52	−4, −**70**, −36	128	−42, −55, −13	−60, 36, −147	134
Sal12 ex vivo	−**3**, −57, −42	−**95**, 0, −62	92	−56, **49**, −115	−26, −**58**, −30	108	−42, −46, −**12**	−58, 11, −**121**	110
Sal13 ex vivo	**43**, −4, −80	−**88**, 22, −111	131	−15, **71**, −49	−48, −**39**, −70	110	3, 52, −**12**	−9, 33, −**143**	131
Sal14 ex vivo	**43**, 35, −45	−**72**, 6, −67	115	2, **63**, −98	8, −**43**, −72	106	−13, 46, −**20**	5, 5, −**140**	120
Sal22 in vivo	**64**, 22, −73	−**53**, 10, −56	117	55, **31**, −64	−11, −**9**, −39	40	−10, 3, −**36**	31, −7, −**133**	96

*Note*: In bold is the angle about the axis about which max/min is measured. The other angles show interaction of degrees of freedom, i.e. the rotations about the other two axes at the maxima and minima of the axis of interest. Positive rotation about *Z* is extension (retraction), positive rotation about *Y* is abduction, positive rotation about *X* is external rotation. Angle values are relative to a null pose in which the proximal femoral ACS is aligned with the acetabular ACS (limb is extended laterally).

**TABLE 2:** Ex vivo and in vivo knee RoM. Euler angles for max and min values are listed in a *Z*, *Y*, *X* format, corresponding to FE, ABAD, LAR

	Max FE	Min FE	Range FE	Max ABAD	Min ABAD	Range ABAD	Max LAR	Min LAR	Range LAR
Sal06 ex vivo	**133**, 24, 5	**5**, −3, 7	128	133, **26**, 5	18, −**27**, 0	53	85, −6, **30**	8, −7, −**35**	65
Sal08 ex vivo	**115**, −34, 13	16, −23, 18	99	49, **16**, −15	95, −**38**, 14	53	54, −6, **33**	26, −5, −**21**	54
Sal22 in vivo	**124**, 17, −17	**10**, −2, −7	115	105, **22**, −18	31, −**15**, 7	37	48, −6, **9**	109, 22, −**22**	32

*Note*: In bold is the angle about the axis about which max/min is measured. The other angles show interaction of degrees of freedom, i.e. the rotations about the other two axes at the maxima and minima of the axis of interest. Positive rotation about *Z* is flexion, positive rotation about *Y* is abduction, positive rotation about *X* is external rotation. Angle values are relative to a null pose in which the proximal tibial/fibular ACS is aligned with the distal femoral ACS (limb is extended laterally).

## 3 RESULTS

### 3.1 Hip

**FIGURE 4:**
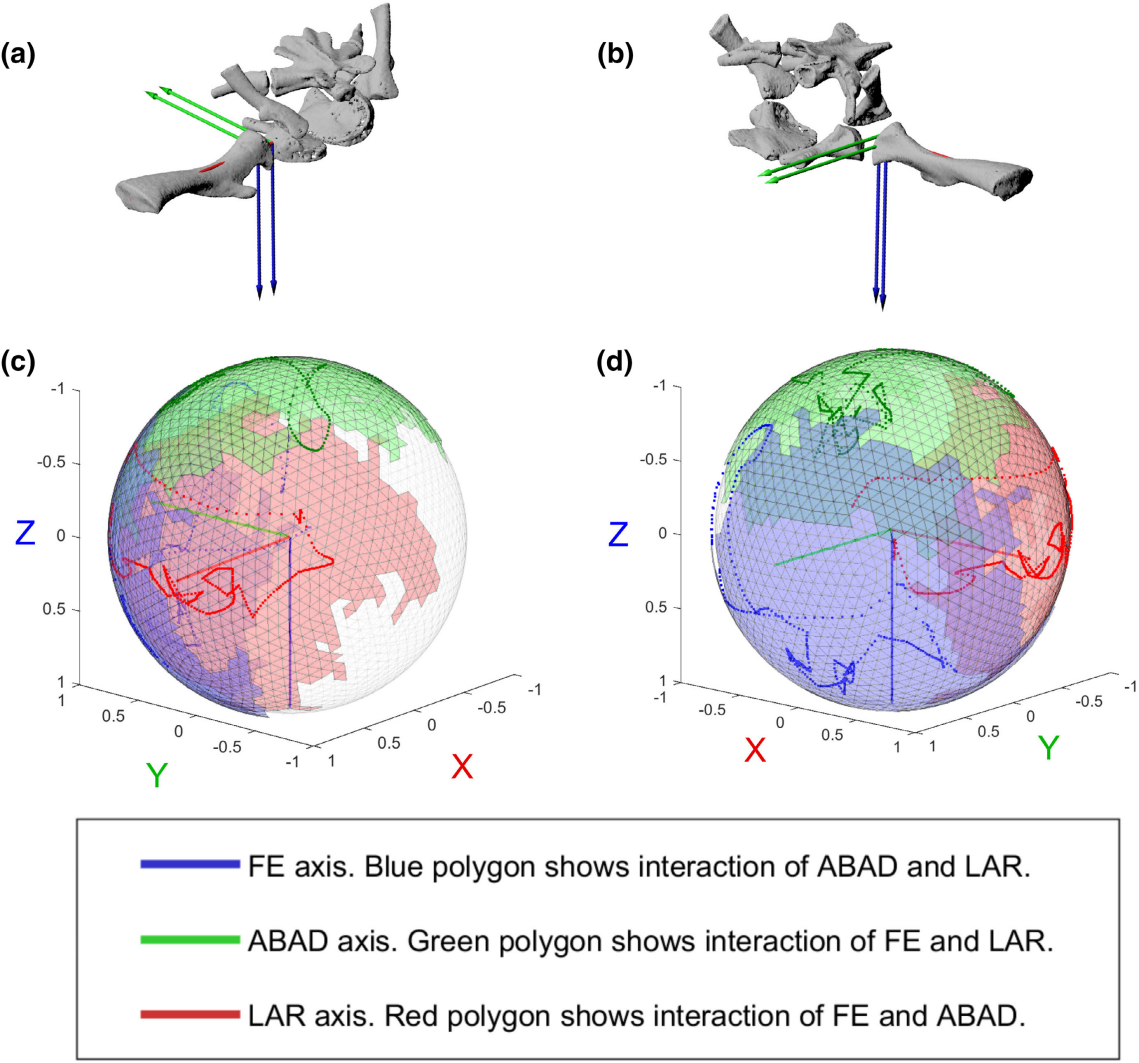
Comparing in vivo and ex vivo hip data using SFPs. SFPs show the excursion of the proximal ACS of the femur, relative to the acetabular ACS. (a) Right hip in anterolateral and (b) posterolateral view; (c, d) SFPs in (c) anterolateral and (d) posterolateral views. Pooled ligamentous RoM data from 3 ex vivo right hips and 1 ex vivo left hip (transformed to right) are shown as shaded polygons, in vivo RoM data during walking are shown as points. Units on axes refer to length of unit vectors of the acetabular ACS axes.

Figure 4 shows the SFPs of pooled data from the ex vivo individuals (shown as shaded polygons) as well as the in vivo data (shown as points on the polygons). The datapoints for the individual ex vivo specimens are shown in Figure S3. Visually, the SFPs can be used to determine the maximum excursions of FE, ABAD and LAR. Movie S2 shows the SFP from all angles. We included all rotational data in these figures, including positions that were translated relative to the null pose, but we did not quantify salamander joint translations in this study. Specific RoM range values and the rotations about the other two axes at which these are achieved are shown in Table 1.

None of the specimens reached the null (reference) pose, in which the proximal femoral ACS was aligned with the acetabular ACS (Table 1). Instead, all possible poses occurred with some degree of internal rotation from the null pose. Anatomically, this internal rotation of the femur enables the morphology of the acetabulum and proximal femur to match so that they both have the longest dimension in the anteroposterior direction (Figure 4a,b, Figure S5).

In ex vivo hip joints, the FE, ABAD and LAR axes all had large ranges of excursion, whereas in the in vivo individual, the ABAD excursion was much smaller than the excursions about the other axes. In vivo ranges fell within the ex vivo ranges, with the in vivo FE range most closely corresponding to the ex vivo ranges (within the variation of ex vivo individuals). However, the in vivo individual achieved greater flexion than the ex vivo individuals. In other words, the in vivo FE range was shifted relative to the ex vivo FE range, exhibiting slightly more extension than flexion, whereas in the ex vivo individuals there was more flexion than extension (Table 1 and Figure 4c).

The in vivo LAR range was a bit less than the ex vivo LAR range, and the in vivo ABAD was about a third of ex vivo ABAD (Figure 4 and Table 1).

In vivo (walking) hip LAR was much more variable across the stride cycle for the hip than it was for the knee (below). In both the ex vivo and in vivo datasets, interaction of degrees of freedom mattered and maxima about one axis were not achieved at 0° rotation about the other two axes.

### 3.2 Knee

**FIGURE 5:**
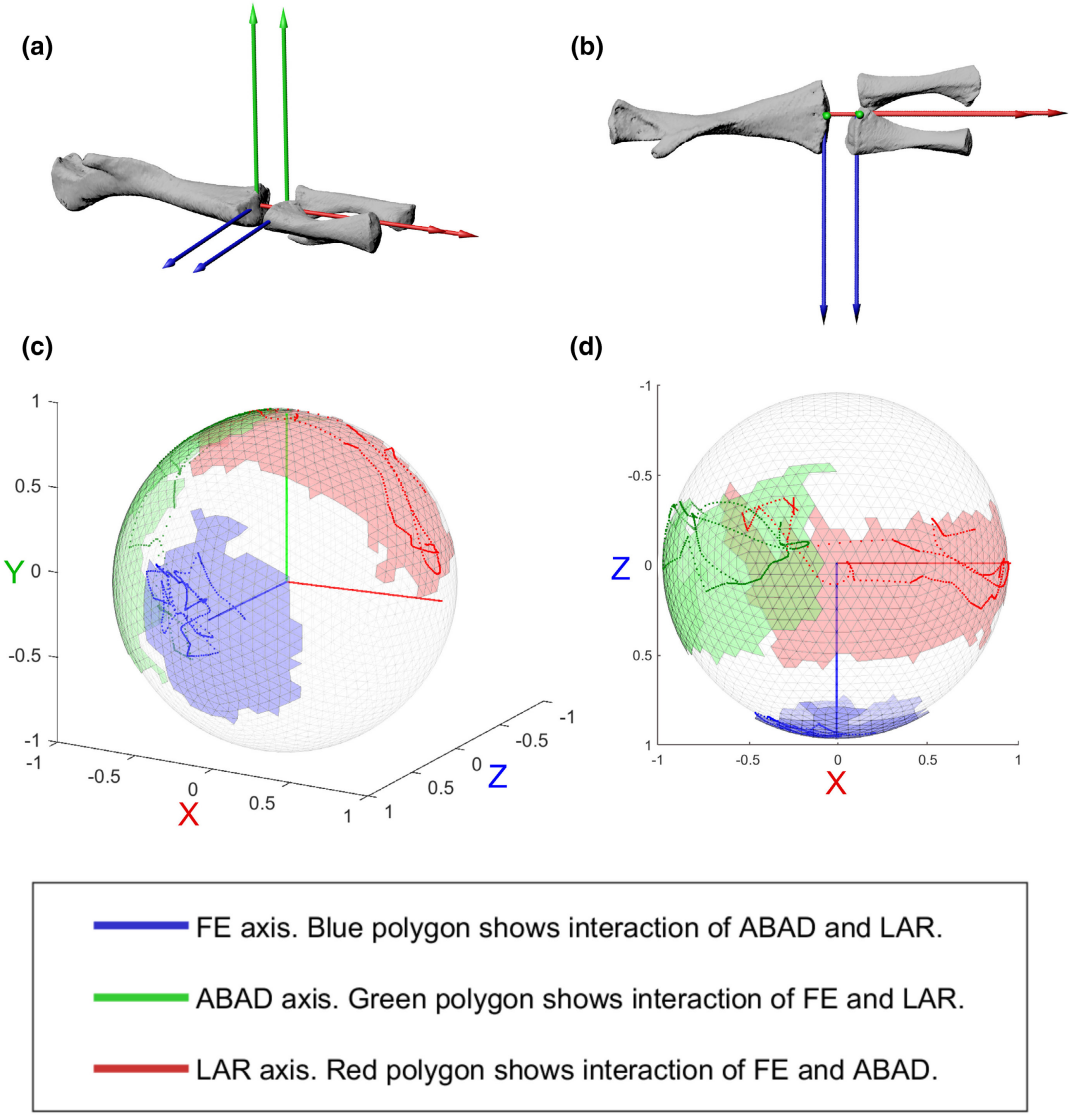
Comparing in vivo and ex vivo knee data using SFPs. SFPs show the excursion of the proximal ACS of the tibia/fibula, relative to the distal ACS of the femur. (a) Right knee, anteroventral view, showing distal femur and proximal tibia/fibula ACSs. (b) Right knee, ventral view, with positive ABAD (*Y*) axis pointing out of page. (c, d) SFPs in (c) anteroventral and (d) ventral views. Pooled ligamentous RoM from two ex vivo individuals are shown as shaded polygons, in vivo RoM during walking is shown as points on the sphere. Units on axes refer to length of unit vectors of the distal femur ACS axes.

Figure 5 shows the SFPs of pooled data from the ex vivo individuals (shown as shaded polygons) as well as the in vivo data (shown as points on the polygons). The datapoints for the individual ex vivo specimens are shown in Figure S4. Movie S3 shows the SFP from all angles. Table 2 shows the in vivo and ex vivo rotational RoM converted to Euler angles. Results for minimum and maximum angles are given for each axis along with the corresponding rotations about the other two axes.

As expected, the greatest RoM of the knee joint in both in vivo and ex vivo individuals occurred about the FE (blue) axis (Figure 5 and Table 2). The amount of excursion about the FE axis is shown by the long dimensions of the red and green polygons in Figure 5. The red polygon shows the interaction of FE and ABAD, and the green polygon shows the interaction of FE and LAR.

The in vivo FE range, maximum flexion, and maximum extension were similar to the ex vivo values. In the in vivo salamander, maximum flexion and extension were achieved with greater internal rotation—in other words, the FE range, while showing similar maxima and minima and range magnitude, was shifted relative to the ex vivo individuals (Table 2, Figure 5c and Movie S3). This meant that some in vivo poses could not be achieved ex vivo (dark green in vivo points in Figure 5c and Movie S3 falling slightly outside of ex vivo polygons).

However, the ex vivo individuals also showed variation between the rotations associated with their greatest FE range. With the ABAD and LAR rotations at or near 0 degrees (like the null pose configuration), the amount of possible FE in salamander 08 was minimal, whereas in salamander 06, a larger FE excursion was possible (Figure S4f). In figures such as these, imagine rotating about the blue (FE) axis only. The excursion of the red axis here is small (short “width” of red polygon in Figure S4f), showing a small FE range when the knee had 0 degrees ABAD and 0 degrees LAR from the null pose. However, if the ABAD and LAR axes are rotated, a much larger FE can be achieved (longest dimension red polygon in Figure S4e,f).

Almost all minima and maxima about the axes were reached when the other two axes were not at the null pose. The patterns of interaction of degrees of freedom were not always consistent between the two individuals; sources of variation between individuals are discussed in the sections below.

The SFP showed that during walking in the representative individual, the most motion of the knee joint occurred about the FE axis. However, motion about the ABAD and LAR axes also contributed to the knee movement that facilitated the sprawling salamander's walking gait, with various combinations of rotations about the FE, ABAD and LAR axes across the stride cycle (Figure 5 and Table 2).

ABAD ranges had similar magnitudes to LAR ranges in both the ex vivo and in vivo individuals. All FE data were positive; hence all of these data points were flexion relative to the null pose, with no extension. 0° of flexion was not a viable pose. This is not surprising because the null pose was a completely straightened limb, so extension (− values for FE) would be a biologically unrealistic hyper‐extension of the knee. Both abduction and adduction relative to the null pose were reached.

## 4 DISCUSSION

### 4.1 New method for joint range of motion experiments

Here, we applied a new experimental rig to investigate joint RoM in salamander hindlimbs. The SFP visualisation method is especially useful because it avoids Gimbal lock problems. In other words, the visual distances on the sphere correspond to differences in joint poses and are not skewed in certain areas as in the Euler angle plots. More details about the applications and benefits of the rig, as well as the new SFP method, can be found in the companion methods paper (Herbst, Eberhard, et al., 2022). Future experiments on salamanders could include the ex vivo load cell data to give insight into passive moments associated with specific movements at the joints. It would be interesting to map out different moment ranges to different postures, and compare these different poses to the poses used in vivo during different points in the stride cycle (and, ideally, in vivo joint moments). Furthermore, lifestyle in salamanders is correlated with the amount of articular cartilage; studies could test the effect of varying amounts of cartilage in different salamander species on loads at the joint (Molnar, 2021).

The Rhino and Matlab software‐based method of assigning ACSs provided a repeatable alternative to other methods of assigning ACSs and can be adopted using other software. Our sensitivity studies are given in Appendix. The point‐fitting method is advantageous for animals like salamanders that have extensive articular cartilage, and our ACS code can easily be altered to adapt the ACS to the joint's morphology.

### 4.2 In vivo and ex vivo joint RoM

We hypothesised that the in vivo RoM would be a subset of ex vivo RoM, operating about the “centre” of the ex vivo RoM rather than near the bounds. Generally, the in vivo RoM fell within the ex vivo RoM, although there were some exceptions in certain poses.

#### 4.2.1 Hip joint

For the hip, there was no consistent trend in relative magnitudes of excursion ranges (e.g., which axis had the most possible motion). In the ex vivo individuals, the greatest range was about the LAR axis, whereas in the in vivo individual the greatest range was about the FE axis. The FE range in the in vivo specimen was contained within the range of the ex vivo specimens (e.g., some ex vivo specimens had lower RoM, some higher), but the in vivo ABAD and LAR were less than in the ex vivo specimens.

When taking into account interaction of degrees of freedom, the variation within ex vivo specimens and between ex vivo and in vivo specimens became even more pronounced. The rotations about the other two axes corresponding to a maximum or minimum at the axis of interest differed between specimens (Table 1). In the cadaveric specimens, the maximum flexion was coupled with more internal rotation than the internal rotation at maximum extension, but in the in vivo data the opposite was true. Therefore, these interactions of the cadaver specimens could not be used to predict the combination of postures used by the salamander in vivo. Note that some of the variation between individuals in the combination of rotations used to achieve maxima/minima about the third axis may be due to sampling differences; some regions of the pose space in certain individuals were sampled less densely (e.g. red polygon Figure S3g). Therefore, the results in Tables 1 and 2 must be taken with some caution, and the overall SFP shapes (especially from the individuals with more homogenous sampling), and the pooled SFPS, can be used to more confidently assess trends in interaction of degrees of freedom.

In the salamander hip joint, the in vivo ABAD range was much less than the cadaver experiments, at about one third of the magnitude of the ex vivo experiments (Table 1). This drastic difference might be caused by the absence of muscles and skin around the hip joints of the cadavers. During the dissections, we observed that removal of skin and muscle at the hip joint greatly increased RoM at the joint. Future studies could test hip RoM with muscle and skin on; we predict that inclusion of these tissues would give a closer correspondence between in vivo and ex vivo data (e.g., Arnold et al., 2014). It is also worth noting here that Arnold et al. (2014) found inter‐individual differences in in vivo RoM, probably related to differing speeds. It would be interesting to rotoscope both another (e.g., faster) stride of this same individual as well as of a different salamander(s) to check the variation amongst in vivo analyses for salamanders. Furthermore, our in vivo dataset only investigated walking on level ground ‐ other behaviours such as swimming and walking over different substrates may utilise different poses, and should be investigated.

In FE, the in vivo RoM was similar to the ex vivo range in salamander 10 (which had a similar size and degree of ossification as the in vivo individual) and salamander 14. Salamander 12 had a much smaller range of FE, and salamander 12 had a greater range (Table 1). The FE range of the in vivo individual was also shifted towards more extension (i.e. retraction), as indicated by the more positive FE values. The in vivo individual achieved the greatest degree of extension relative to the null pose compared to all of the ex vivo individuals (Table 1). This is also shown in the SFPs (Figure 4d and Movie S2), where the red dots (in vivo data) exceed the red polygon (ex vivo data) (recall that rotation about the blue FE axis causes movement of the red and green axis endpoints).

Analysing the SFPs (Figure 4 and Movie S2) gives more insight into the overall hip RoM including interactions of degrees of freedom. Although the maximal internal rotation (maximum negative LAR) of the in vivo individual fell within the range of the ex vivo individuals, some poses used during walking fell outside of the ex vivo datapoints. This is shown in Figure 4d, where blue points tracing the in vivo excursion in Figure 4d exceed the blue ex vivo patches on the top left side of the sphere (recall that blue axis excursion can be caused by rotation around the green (ABAD) and red (LAR) axes). These poses were achieved when the femur was maximally internally rotated and extended (retracted) during walking, during the end of stance phase just before toe‐off. The regions where in vivo poses fell outside of the ex vivo pose space could be due to individual morphological variations between specimens. Figure S5 shows the osteological variation, although it is important to note that differences in joint morphology could also have been present at the cartilage level, which was not segmented in our scans. Furthermore, it could be possible that the ex vivo joint RoM space was slightly undersampled at the margins of the pose space; due to the finite number of specimens we had, we chose to be conservative with the torque threshold used in sampling. Larger thresholds could be tested with a larger dataset.

The ex vivo specimens also varied in their joint RoM. This could also be due to experimental variation in the specimens. We reduced sampling biases by using the method's live feedback to check there were no gaps in poses covered, but it is nonetheless possible that due to an error we did not cover all possible poses, for example for salamander 12, which shows a lower FE and LAR range than the other specimens (Table 1). Another source of the variation could be variation in dissection. For the hips, we tried to cut all muscle bellies to prevent muscles from restricting RoM. However, due to the fragility of the hip joint (compared to the knee), we had to keep some sections of muscle origins and insertions intact to prevent the joint capsule from rupturing. Perhaps salamander 12 had more muscle left on it that restricted movement. Such dissection or sampling biases could be reduced when working on larger, less fragile individuals, where joint damage is not as much of a risk.

The variation in mobility between the ex vivo specimens could also be attributable to differences in pelvis or femoral morphology. When examining the microCT scans, we noticed a pathological bump on the distal femur of salamander 13 (similar in appearance to the growths described in early tetrapods in Herbst et al. 2019) (Figure S5F). The pelvis was also the most ossified of all individuals. Salamanders can vary in degree of ossification of the pubo‐ischium (Francis, 1934). We included the highly ossified, pathological individual in the hip analyses because the pathological growths were on the distal femur (Figure S5). The hip RoM of the highly ossified individual was not restricted relative to other salamanders (Table 1), which is to be expected since the extra bone did not appear to constrict the hip joint. The SFP for salamander 13 (pathological specimen) was more similar to that of salamander 10 than either was to salamander 12 (Figure S3). Using the null pose as a reference, salamander 12's RoM was shifted compared to the other two salamanders. Salamander 10 and salamander 13 could achieve poses with 0° FE and 0° ABAD (in other words, the endpoint of the red axis was inside the polygon). For salamander 12, the combination of 0° FE and 0° ABAD could not be reached. Additionally, the RoM of FE was lower than in the other two ex vivo specimens. Variation in hip joint RoM cannot be attributable to general differences in size or variation in pelvis ossification, because salamanders 10 and 22 were the smallest and least ossified individual (no bony ossification between ilium and ischium), and the SFP of salamander 10 most resembled salamander 13, the most ossified specimen. Out of the ex vivo specimens, salamander 10 had the largest ABAD and LAR ranges, perhaps because the bones were less restrictive in these motions. However, the FE range was largest in salamander 13. Future studies could investigate the effects of more subtle morphological differences, as well as variation in soft tissue anatomy, on joint RoM.

#### 4.2.2 Knee joint

At the knee joint, for both in vivo and ex vivo experiments, the most motion occurred about the FE axis in both ex vivo and in vivo individuals. LAR and ABAD ranges were much less than FE, and were of roughly similar magnitudes within individuals. These general patterns were consistent across ex vivo and in vivo experiments, therefore studies could be used to qualitatively predict which axis of rotation has the largest excursions in vivo. This knowledge is useful for rotoscoping setups, where the *Z*‐axis should be assigned to the axis with the most motion (given a *Z*‐*Y*‐*X* rotation order), to reduce difficulties due to gimbal lock.

The FE range in the in vivo specimen was within the range of variation in FE ranges observed for ex vivo specimens, and the ABAD and LAR ranges were lower than in both ex vivo individuals, corresponding to our prediction based on previous studies (e.g., Arnold et al., 2014) that the in vivo range of RoM would be a subset of ex vivo RoM.

Comparison of the SFPs (Figure 5) reported in the above sections shows that overall, a wider range of knee poses was achieved in cadaver experiments than the range of poses covered during walking. However, when only looking at ranges about the three axes (Table 2), in vivo RoM is not necessarily a subsection of ex vivo RoM; the in vivo FE range (salamander 22) was less than that in ex vivo salamander 6 but greater than in ex vivo salamander 8. Contrastingly, for both ABAD and LAR ranges, the ranges were lower during walking (salamander 22, in vivo) than in both ex vivo specimens. The SFP (Figure 5) shows that the in vivo ROM is not centred within the ex vivo dataset, but more studies with a larger sample size and examining the variation between strides in a single individual are needed to test for reliable trends between in vivo and ex vivo joint RoM (as was done in Manafzadeh et al., 2021).

During maximum flexion of the knee (around mid‐stance), as well as maximum extension, the in vivo individual had more negative LAR (internal rotation) than in the ex vivo individuals, in which maximum flexion was associated with external rotation (Table 2). Figure 5d and Video S3 also show that in the regions of high flexion, there were some poses in the in vivo individual that fell outside of the ex vivo pose space (see red and green dots; in vivo *walking data* were outside of the ex vivo polygons, specifically at areas of high flexion, i.e. positive rotation about blue axis). As for the hip, this variation could be due to individual morphological variation (see Figure S6 for skeletal comparisons of the specimen), including cartilage. Future studies could investigate links between joint articular (i.e. cartilage) structure and joint mobility variation. Another reason for the differences in poses of the knee specifically could be that in the in vivo experiments the tibia and fibula could move relative to each other whereas in the ex vivo experiments their relative positions were fixed (a necessity for data collection due to the small size of these specimens).

Variation between the ex vivo specimens could also be due to variations in sampling. Joint range of motion variation due to dissection differences are unlikely at the knee because for this joint it was straightforward to remove all of the muscles (relative to the hip joint), and damage at the joint was also more easily detected at the knee. Therefore, we suspect the differences between salamander 6 and salamander 8 probably were not due to variation in dissections.

### 4.3 Comparisons with other studies

Using video data, Pierce et al. (2020) found that a in vivo hip protraction/retraction (FE) RoM of 100° and a knee FE RoM of 60°, whereas we found an in vivo hip FE RoM of 117° and a knee FE RoM of 115° (Tables 1 and 2). Note that for the knee, individuals salamander 06 and salamander 08 were used in both studies. The large difference between the knee FE RoM is likely due to the difficulties of measuring joint angle excursions from light videos—especially for the knee, poses with high knee flexion might be difficult to detect from lateral and dorsal light videos in sprawling hindlimbs.

Our in vivo data are in general in qualitative and quantitative agreement with other analyses of limb joint RoM in salamanders. Edwards (1989) recognised the importance of both LAR and FE of the hip to facilitating the sprawling gait of salamanders, which is corroborated by our in vivo rotoscoping analysis of the hip joint. The ranges of hip ABAD and LAR from our in vivo *S. salamandra* experiments fall within one standard deviation as reported for femur ABAD and LAR values in the newt *Pleurodeles waltl* (Karakasiliotis et al., 2013); but our hip FE values are slightly larger than those that they reported, perhaps due to variation between species or individuals.

As in *Pleurodeles waltl*, we found that knee FE shows a wide excursion over the stride cycle (Karakasiliotis et al., 2013). The in vivo data for the newt *Pleurodeles* reported in Nyakatura et al. (2019) cannot be directly compared to our data, because only values during stance phase were reported in that study. Furthermore, for hip FE, only femoral retraction (from a laterally extended) null pose was reported. This accounts for the lower hip FE and LAR values in their rotoscoped salamander data relative to our rotoscoping data (which included the swing phase and femoral protraction, which occurs relative to the null pose). Additionally, the study reported an inverse relationship between femoral LAR and FE, with more sprawling taxa using relatively more LAR, and femoral LAR in salamanders exceeding femoral FE during the stance phase (ground contact) of locomotion. However, when the whole stride is taken into account, and both femoral protraction and retraction are measured, our data showed that FE was larger than LAR in the sprawling salamander, and this was also the case for the study by Karakasiliotis et al. (2013). Excluding protraction data could bias the comparison between different animals, because the null pose could occur at different regions of the overall motion space in different animals, based on their gait and joint morphology. Therefore, it would be interesting to see the same comparisons of LAR and FE in different taxa shown in Nyakatura et al. (2019), but including protraction and swing‐phase data, to see if the relative trends between taxa (inverse relationship between magnitudes of FE and LAR) still hold outside of stance phase.

### 4.4 Implications for future studies

Studies often use gaits of extant taxa as a hypothesis, model or analogue/homologue for the gait used in extinct taxa. Such locomotor hypotheses can be tested by comparing fossil joint RoM to in vivo or ex vivo RoM in the extant taxon, to determine if the fossil could have achieved the joint poses required to achieve the gait. Our goal was to examine the differences between the joint RoM obtained from ligament‐only cadaver experiments and the joint RoM employed by Fire Salamanders during walking. Additionally, our motivation for testing the ligament‐only salamander joint RoM (rather than including muscles and skin in the cadaver analysis) was to more closely mimic the situation in fossils, where we usually only have osteological features preserved. However, our experiments showed that the best dataset to use for comparison with fossil joint RoM is the in vivo dataset of the extant animal, if it exists. This might seem counter‐intuitive, but fossil osteological RoM studies are usually based on excluding certain poses. Therefore, the possible poses should be checked against the poses required for the actual gait we are interested in (i.e., the in vivo range of the animal), rather than comparing the fossil osteological joint RoM to the osteological joint RoM in extant animals. Arnold et al. (2014) also observed large differences in ex vivo and in vivo joint RoM in the iguana, and also came to the conclusion that in vivo locomotor analysis of extant taxa (rather than just using osteological RoM data) is important for inferring gait in extinct taxa.

Using only the ex vivo salamander dataset as comparative data to determine if a fossil moved with a salamander‐like walk could lead to the wrong conclusions. For example, assume a scenario in which the in vivo joint RoM during a certain behaviour in an extant animal is a subset of the poses reached in the ex vivo individuals. The hip ABAD is an instructive example from our experiments. During walking, the salamander had a much lower range (almost a third of the range) of ABAD than was possible in the cadaver ligament‐only experiments. Now imagine an analysis of the osteological hip joint RoM of an early tetrapod finding that the possible ABAD was half of that in the salamander ex vivo experiments. One might conclude that the fossil had different in vivo joint RoM than a salamander for a certain behaviour. However, the salamander's in vivo RoM for normal locomotion might fall within the fossil's osteological joint RoM, which would mean a salamander‐like sprawling gait could have been possible. Comparing fossil osteological RoM with salamander osteological or other ex vivo (e.g. ligamentous) RoM may lead to overestimates of the amount of joint RoM required to move like a salamander; more studies are required to test the relationships of osteological, ligamentous and in vivo RoM. With advances in scanning technology and scientific rotoscoping, using in vivo experiments of extant animals for comparisons with extinct animals could also reduce the number of animals sacrificed during ex vivo experiments.

However, ex vivo RoM data can still be useful; other studies have shown similar patterns between in vivo and ex vivo RoM in archosaur hindlimbs (Manafzadeh et al., 2021). Our dataset also shows that some general trends are shared between in vivo and ex vivo salamander RoM, for example the maximum rotation occurring around the FE axis of the knee. Further studies with larger sample sizes are needed to investigate whether similar relationships between in vivo and ex vivo RoM found in archosaurs hold true in salamanders. Consistent trends between ex vivo and in vivo patterns across tetrapods could have important implications for addressing questions of early tetrapod evolution, such as whether certain early tetrapods were more forelimb‐ or hindlimb‐ driven. Furthermore, it would be interesting to test if ontogenetic changes in locomotion are reflected in ex vivo RoM of specimens at different ontogenetic stages.

The ex vivo and in vivo datasets for the salamander hip and knee joints demonstrate the importance of considering interaction between degrees of freedom. The maximum and minimum values about one axis depend on the rotations about the other two axes. Disregarding this interaction by only characterising maxima and minima can lead to both over‐ and underestimates of the RoM. Overestimates occur by assuming all three maxima about the rotational axes are achievable in the same pose (Kambic et al., 2014, 2017; Manafzadeh & Padian, 2018). Underestimates occur if the null pose rotations about two axes limit the maximal excursions of the axis of interest. Quantifying the possible combinations of poses is important when comparing fossil joint RoM to an extant animal's joint RoM to infer gait. For example, determining whether a gait and foot position was possible depends not only on attaining a specific degree of FE at the knee and hip but also whether the joint can attain the necessary rotations about the other two axes when in that specific degree of FE.

Gait inferences in fossils are inherently difficult due to all of the soft tissue and behavioural data we are missing. However, the field has seen many advancements that enable us to best use the lines of evidence we have. Fossil joint biomechanics have progressed from qualitative descriptions based on the joint anatomy (Shubin et al., 2006) to exclusion‐based, digital osteological RoM studies (Pierce et al., 2012) to rigorous, automated testing of interaction of degrees of freedom in osteological RoM (Manafzadeh & Padian, 2018), and combining trackways, robotics, full body simulations and comparative in vivo data to infer gait (Nyakatura et al., 2019). All of these new methods are bringing us closer to conducting the most comprehensive analyses of locomotion in extinct and extant animals. Our study's contributions to the field's wide efforts are novel experimental and analytical methods to quantify salamander RoM, and a detailed dataset of in vivo and ex vivo joint RoM of Fire Salamander hip and knee joints. Our dataset reveals how interaction of rotations about anatomical axes facilitate a sprawling walk in salamanders, and can act as comparative data for testing the possibility of this gait in early tetrapods.

## 5 CONCLUSION

Here, we summarise the main findings of our study, and how they relate to our initial aims.

- *Aim 1*: We successfully implemented our joint RoM rig and SFP method (Herbst, Eberhard, et al., 2022), capturing a detailed dataset of the hindlimb joint range of motion in *S. salamandra* including interaction of degrees of freedom.
- *Aim 2*: The SFP approach enabled intuitive comparisons between in vivo (during walking) and ex vivo (ligamentous) ROM data.
  - In the hip, ex vivo FE, LAR and ABAD had similar ranges, whereas in the in vivo salamander, the ABAD range had the lowest excursion (much lower than ex vivo ABAD), followed by LAR (a bit lower range than ex vivo), and a similar FE range to ex vivo.
  - The in vivo hip FE range was slightly biased towards extension rather than flexion, whereas for the ex vivo individuals there was much greater flexion than extension.
  - In the salamander knee, there was a strong correspondence between in vivo and ex vivo FE range, maxima and minima, suggesting that cadaver studies of ROM may give reasonable predictions of the in vivo FE range for this joint.
  - However, the in vivo FE range was shifted (more internal rotation) relative to the ex vivo ranges. Further studies are needed to determine whether this is a consistent trend, or due to inter‐individual variation.
  - Knee in vivo ABAD and LAR were a subset of ex vivo poses, meaning that in these rotational axes, the joints operate well within their limits during walking.
  - In the knee, some similar patterns were observed in vivo and ex vivo: in all specimens, FE was the greatest range, and within an individual, ABAD and LAR ranges were similar.
  - Interactions of degrees of freedom are important; maximum and minimum joint rotations were rarely reached close to the null pose.
- *Aim 3*: The pose space of *S. salamandra* knee and hip joints reported here can be used in future studies as a reference dataset comparing fossil RoM to joint RoM in *S. salamandra*. This dataset was used (Herbst, Manafzadeh, & Hutchinson, 2022) to investigate whether salamander‐like hindlimb kinematics were possible in the early tetrapod *Eryops*.

## AUTHOR CONTRIBUTIONS

E.A.E. and E.C.H. conducted the ex vivo experiments and curated the code and data. E.C.H. wrote the paper, made the figures, dissected the specimens, conducted the scientific rotoscoping, and assigned ACSs. E.A.E. wrote the Matlab code, with adjustments from E.C.H. for ACS definitions. C.T.R. and J.R.H. acquired funding and supervised the project, and J.R.H. also provided specimens and x‐ray videos. All authors edited the manuscript.

## FUNDING INFORMATION

This work was funded by a European Research Council Starting Grant PIPA338271 and the Natural Environment Research Council NE/K004751/1.

## CONFLICT OF INTEREST

The authors declare no competing interests.

## Supporting information


MovieS1

MovieS2

MovieS3

DataS1

Figure S1

## Figures and Tables

**FIGURE A1:**
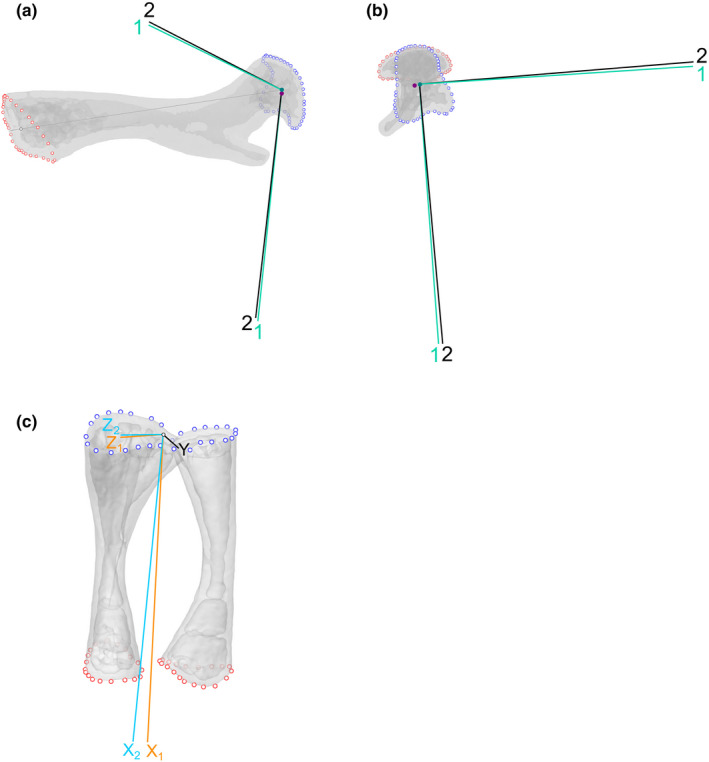
(a, b) Sensitivity analyses comparing different approaches to calculating the ACS of the proximal femur in (a) anterior view; (b) view of proximal bone surface. The purple dot marks the origin calculated by fitting a geometric primitive to the segmented cartilage of another individual, and transferring this origin to this individual using CloudCompare. The turquoise dot is the origin calculated by using the mean of the points (in blue) placed around the perimeter of the proximal bone. The axes can be calculated based on either the proximal bone morphology (1, in turquoise, used in this paper) or the distal bone morphology (2, in black). The *X*‐axis (LAR) remains the same in these methods, but the *Y* and *Z* axes differed very slightly. (c) Axes recalculations to produce ACS with orthogonal axes. To make the axes orthogonal, either the *X*‐axis can be recalculated (blue) or the *Z*‐axis can be recalculated (orange). We recalculated the *Z*‐axis for our analysis (Z1), so that the LAR axis is kept in a more anatomically intuitive position (X1).

## Data Availability

3D models (.stls) of the salamander bones, as well as the ex vivo motion capture data, are available on Figshare (https://doi.org/10.6084/m9.figshare.20060564 and https://doi.org/10.6084/m9.figshare.20060561). Relevant code for this research work is stored in GitHub: (https://github.com/evaherbst/Joint_RoM_SFP) and has been archived within the Zenodo repository: (https://doi.org/10.5281/zenodo.6914547).

## APPENDIX A

### A.1 Dissections and Rigging

Prior to dissection, each frozen specimen was placed in warm water for a few hours to thaw. Then, we skinned the specimen and removed the limb of interest. Only one knee or hip joint could be used per limb, because the other joint was destroyed in the rigging procedure (see below; rigging is the process of securing the elements to the rig). We filled a small dish with Ringer's solution and performed part of the dissection with the limb pinned to this dish under a dissecting microscope. For the more difficult cuts close to the fragile hip joint capsule, we removed the specimen from the dish and microscope, to better manipulate the specimen to precisely determine the depth of the incisions. To keep the tissue hydrated, we submerged the specimen in Ringer's solution every few minutes during dissections (Manafzadeh, 2020).

We glued the proximal and distal bones to acrylic plates with methacrylate glue (Devcon Devweld 530) and wire (Figure S2). We then waited 20 min for the glue to dry, occasionally spraying the tissue during this period to keep it hydrated. The acrylic plates were created using a laser cutter; all plates were created from the same cutting template and therefore had the same dimensions (~30 mm), to facilitate data transformations. We secured the bone to the plate using wire and glue, ensuring that no glue or wire came in contact with the joint. For the hips (which were more difficult to rig), we sometimes attached a small piece of aluminium foil to shield the joint area from the glue.

### A.2 ACSs compared to other studies

Our acetabular ACSs did not follow the conventions proposed by Kambic et al. (2014), because our null pose and axes directions were defined to enable SFP visualisation (see “Data Visualisation” section). For the SFP null pose, the femur was extended laterally (to enable a more intuitive visualisation of the experiments). Furthermore, salamanders are sprawling animals, so the directions of motion at the hip (FE, ABAD and LAR) relative to the body orientations (dorsoventral, mediolateral and anteroposterior directions) are different from parasagittal locomotors, such as the guineafowl investigated by Kambic et al. (2014). For example, the hip's FE axis in guineafowl is oriented roughly mediolaterally (Kambic et al., 2014, figure 11a), whereas for salamanders, the hip's FE axis should be oriented roughly dorsoventrally (Figure 2a). The axes of the acetabular ACS were defined as follows for the right acetabulum and are illustrated in Figure 2. The *Z*‐axis (FE) pointed ventrally; positive rotation about the *Z*‐axis was extension (retraction) at the hip. The *Y*‐axis (ABAD) pointed caudally; positive rotation about the *Y*‐axis was abduction at the hip. The *X*‐axis (LAR) pointed laterally; positive rotation about the *X*‐axis was external rotation.

### A.3 ACS sensitivity analyses

#### A.3.1. Point placement versus geometric primitives

We conducted a sensitivity analysis to test how the origin of a sphere fitted to the cartilaginous surface of the proximal femur compared to our point placement method. We segmented the femoral head of the specimen with the clearest cartilage, and fit a sphere to the cartilage using geometric shape‐fitting code (Bishop et al., 2021). We then used CloudCompare software to fit this geometric primitive to another specimen (specimen 22). For that specimen, we then also calculated the ACS origin based on our point‐ fitting method. The origins obtained from two methods were only 0.24 mm apart (within 8.4%, given the radius of the fitted sphere was 2.87 mm) (Figure A1). We also tested inter‐user bias by having two users independently place points on the same salamander femur. The proximal origins differed only by 0.029 mm versus a maximum tibia length of about 6 mm (inter‐observer variation of 0.4% of maximum tibia length).

#### A.3.2. Femoral ACS calculation

The proximal femoral ACS could be calculated based either on the proximal or distal bone morphology. Other studies have used the distal femur morphology to calculate the proximal femoral ACS, by using the distal femur morphology to define a distal *Z* (FE) axis. The proximal *Y*‐axis and the proximal *Z*‐axis were calculated from this distal *Z*‐axis (Gatesy et al., 2022; Kambic et al., 2014, 2017; Manafzadeh & Padian, 2018). As a sensitivity analysis, we compared both methods. We found that for salamanders, calculating the *Z* (FE) axis based on the proximal femur morphology or distal femur morphology produced similar axes (Figure A1). Our method of using the proximal morphology is more intuitive, because the proximal morphology of the femur is more relevant to hip joint RoM than the distal morphology is.

#### A.3.3. Point‐based long axis versus axis of least inertia

Kambic et al. (2014) used the axis of least inertia to calculate the LAR axis for the proximal ACS of the guineafowl tibiotarsus. Similar to salamander bones, fitting geometric primitives to the proximal surface of the guineafowl tibiotarsus was not practical due to flat morphology. To test how our point‐based calculation compared to the axis of least inertia, we calculated the axis of least inertia for one individual's tibia and fibula (salamander 06) with Meshlab software v1.3.3 (Cignoni et al., 2008). Using the point placement method to calculate the long axis of the tibia and fibula is comparable to the long axis defined as the axis of least inertia; the difference between the two methods was only 0.393°. The point‐based method is preferable for this study because it is based on the anatomy of the bone surfaces and therefore more intuitive to understand.

## References

[joa13738-bib-0001] Arnold, P. , Fischer, M.S. & Nyakatura, J.A. (2014) Soft tissue influence on ex vivo mobility in the hip of iguana: comparison with in vivo movement and its bearing on joint motion of fossil sprawling tetrapods. Journal of Anatomy, 225, 31–41. 10.1111/joa.12187 24762236 PMC4089344

[joa13738-bib-0002] Ashley‐Ross, M. (1994) Hindlimb kinematics during terrestrial locomotion in a salamander (Dicamptodon Tenebrosus). The Journal of Experimental Biology, 193, 255–283. 9317755 10.1242/jeb.193.1.255

[joa13738-bib-0003] Bishop, P.J. , Cuff, A.R. & Hutchinson, J.R. (2021) How to build a dinosaur: musculoskeletal modeling and simulation of locomotor biomechanics in extinct animals. Paleobiology, 47, 1–38. 10.1017/pab.2020.46

[joa13738-bib-0004] Brainerd, E.L. , Baier, D.B. , Gatesy, S.M. , Hedrick, T.L. , Metzger, K.A. , Gilbert, S.L. & Crisco, J.J. (2010) X‐ray reconstruction of moving morphology (XROMM): precision, accuracy and applications in comparative biomechanics research. Journal of Experimental Zoology. Part A, Ecological Genetics and Physiology, 313, 262–279. 10.1002/jez.589 20095029

[joa13738-bib-0005] Cignoni, P. , Callieri, M. , Corsini, M. , Dellepiane, M. , Ganovelli, F. & Ranzuglia, G. (2008) MeshLab: an open‐source mesh processing tool. In: Eurographics Italian chapter conference. Salerno: The Eurographics Association, pp. 129–136.

[joa13738-bib-0006] Edwards, J.L. (1989) Two perspectives on the evolution of the tetrapod limb. American Zoologist, 29, 235–254. 10.1093/icb/29.1.235

[joa13738-bib-0007] Francis, E.T.B. (1934) The anatomy of the salamander, Vol. 381. London: Oxford University Press. 10.1038/136087a0

[joa13738-bib-0008] Gatesy, S. , Manafzadeh, A. , Bishop, P. , Turner, M. , Kambic, R. , Cuff, A. et al. (2022) A proposed standard for quantifying 3‐D hindlimb joint poses in living and extinct archosaurs. Journal of Anatomy, 241, 101–118. 10.1111/joa.13635 35118654 PMC9178381

[joa13738-bib-0009] Gatesy, S.M. , Baier, D.B. , Jenkins, F.A. & Dial, K.P. (2010) Scientific rotoscoping: a morphology‐based method of 3‐D motion analysis and visualization. Journal of Experimental Zoology. Part A, Ecological Genetics and Physiology, 313, 244–261. 10.1002/jez.588 20084664

[joa13738-bib-0010] Grood, E.S. & Suntay, W.J. (1983) A joint coordinate system for the clinical description of three‐dimensional motions: application to the knee. Journal of Biomechanical Engineering, 105, 136–144. 10.1115/1.3138397 6865355

[joa13738-bib-0011] Haering, D. , Raison, M. & Begon, M. (2014) Measurement and description of three‐dimensional shoulder range of motion with degrees of freedom interactions. Journal of Biomechanical Engineering, 136, 1–7. 10.1115/1.4027665 24828544

[joa13738-bib-0012] Herbst, E.C. , Doube, M. , Smithson, T.R. , Clack, J.A. & Hutchinson, J.R. (2019) Bony lesions in early tetrapods and the evolution of mineralized tissue repair. Paleobiology, 45, 676–697. 10.1017/pab.2019.31

[joa13738-bib-0013] Herbst, E.C. , Eberhard, E.A. , Hutchinson, J.R. & Richards, C.T. (2022) Spherical frame projections for visualizing joint range of motion, and a complementary method to capture mobility data. Journal of Anatomy. 10.1111/joa.13717 PMC9482700 35819977

[joa13738-bib-0014] Herbst, E.C. , Manafzadeh, A.R. & Hutchinson, J.R. (2022) Multi‐joint analysis of pose viability supports the possibility of salamander‐like hindlimb configurations in the Permian tetrapod Eryops megacephalus. Integrative and Comparative Biology, 1–13. 10.1093/icb/icac083 PMC9405718 35687000

[joa13738-bib-0015] Hutson, J.D. & Hutson, K.N. (2012) A test of the validity of range of motion studies of fossil archosaur elbow mobility using repeated‐measures analysis and the extant phylogenetic bracket. Journal of Experimental Biology, 215, 2030–2038. 10.1242/jeb.069567 22623191

[joa13738-bib-0016] Hutson, J.D. & Hutson, K.N. (2013) Using the American alligator and a repeated‐measures design to place constraints on in vivo shoulder joint range of motion in dinosaurs and other fossil archosaurs. Journal of Experimental Biology, 216, 275–284. 10.1242/jeb.074229 22972888

[joa13738-bib-0017] Kambic, R.E. , Roberts, T.J. & Gatesy, S.M. (2014) Long‐axis rotation: a missing degree of freedom in avian bipedal locomotion. Journal of Experimental Biology, 217, 2770–2782. 10.1242/jeb.101428 24855675

[joa13738-bib-0018] Kambic, R.E. , Roberts, T.J. & Gatesy, S.M. (2017) 3‐D range of motion envelopes reveal interacting degrees of freedom in avian hind limb joints. Journal of Anatomy, 231, 906–920. 10.1111/joa.12680 28833095 PMC5696129

[joa13738-bib-0019] Karakasiliotis, K. , Schilling, N. , Cabelguen, J.M. & Ijspeert, A.J. (2013) Where are we in understanding salamander locomotion: biological and robotic perspectives on kinematics. Biological Cybernetics, 107, 529–544. 10.1007/s00422-012-0540-4 23250621

[joa13738-bib-0020] Kawano, S.M. & Blob, R.W. (2013) Propulsive forces of mudskipper fins and salamander limbs during terrestrial locomotion: implications for the invasion of land. Integrative and Comparative Biology, 53, 283–294. 10.1093/icb/ict051 23667046

[joa13738-bib-0021] Knorlein, B.J. , Baier, D.B. , Gatesy, S.M. , Laurence‐Chasen, J.D. & Brainerd, E.L. (2016) Validation of XMALab software for marker‐based XROMM. Journal of Experimental Biology, 219, 3701–3711. 10.1242/jeb.145383 27655556

[joa13738-bib-0022] Manafzadeh, A.R. (2020) A practical guide to measuring ex vivo joint mobility using XROMM. Integrative Organismal Biology, 2, 1–12. 10.1093/iob/obaa041 PMC7810577 33791578

[joa13738-bib-0023] Manafzadeh, A.R. & Gatesy, S.M. (2020) A coordinate‐system‐independent method for comparing joint rotational mobilities. The Journal of Experimental Biology, 223, 1–8. 10.1242/jeb.227108 32747453

[joa13738-bib-0024] Manafzadeh, A.R. , Kambic, R.E. & Gatesy, S.M. (2021) A new role for joint mobility in reconstructing vertebrate locomotor evolution. Proceedings of the National Academy of Sciences of the United States of America, 118, 8–10. 10.1073/pnas.2023513118 PMC7896293 33558244

[joa13738-bib-0025] Manafzadeh, A.R. & Padian, K. (2018) ROM mapping of ligamentous constraints on avian hip mobility: implications for extinct ornithodirans. Proceedings of the Royal Society B, 285, 20180727. 29794053 10.1098/rspb.2018.0727 PMC5998106

[joa13738-bib-0026] Molnar, J.L. (2021) Variation in articular cartilage thickness among extant salamanders and implications for limb function in stem tetrapods. Frontiers in Ecology and Evolution, 9, 671006.

[joa13738-bib-0027] Nyakatura, J.A. , Melo, K. , Horvat, T. , Karakasiliotis, K. , Allen, V.R. , Andikfar, A. et al. (2019) Reverse‐engineering the locomotion of a stem amniote. Nature, 565, 351–355. 10.1038/s41586-018-0851-2 30651613

[joa13738-bib-0028] Pierce, S.E. , Clack, J.A. & Hutchinson, J.R. (2012) Three‐dimensional limb joint mobility in the early tetrapod Ichthyostega. Nature, 486, 523–526. 10.1038/nature11124 22722854

[joa13738-bib-0029] Pierce, S.E. , Lamas, L.P. , Pelligand, L. , Schilling, N. & Hutchinson, J.R. (2020) Patterns of limb and epaxial muscle activity during walking in the fire salamander, *Salamandra salamandra* . Integrative Organismal Biology, 2, 1–20. 10.1093/iob/obaa015 PMC7671131 33791558

[joa13738-bib-0030] Ren, L. , Butler, M. , Miller, C. , Paxton, H. , Schwerda, D. , Fischer, M.S. et al. (2008) The movements of limb segments and joints during locomotion in African and Asian elephants. Journal of Experimental Biology, 211, 2735–2751. 10.1242/jeb.018820 18723530

[joa13738-bib-0031] Schaeffer, B. (1941) The morphological and functional evolution of the tarsus in amphibians and reptiles. Bulletin of the American Museum of Natural History, 78, 395–472.

[joa13738-bib-0032] Shubin, N.H. , Daeschler, E.B. & Jenkins, F.A. (2006) The pectoral fin of Tiktaalik roseae and the origin of the tetrapod limb. Nature, 440, 764–771. 10.1038/nature04637 16598250

