# Supplementary material for: In vivo and ex vivo range of motion in the fire salamander Salamandra salamandra
Source: J Anat. 2022 Aug 20;241(4):1066–82. doi: 10.1111/joa.13738 (PMC9482696; doi:10.1111/joa.13738)
Supplement: Supplementary file 4 — DataS1 [file JOA-241-1066-s004.docx]

Due to size restrictions, the motion capture data and code could not be uploaded directly. Please find a link to the Matlab code and data here:

motion capture data:

<https://www.dropbox.com/s/oomyvotz8hovplr/Data%20for%20paper.zip?dl=0>

Matlab code :

<https://www.dropbox.com/s/kakuhzhsuxrwc7f/Joint_RoM_SFP-main.zip?dl=0>
