# Supplementary material for: In vivo and ex vivo range of motion in the fire salamander Salamandra salamandra
Source: J Anat. 2022 Aug 20;241(4):1066–82. doi: 10.1111/joa.13738 (PMC9482696; doi:10.1111/joa.13738)
Supplement: Supplementary file 5 — Figure S1 [file JOA-241-1066-s003.docx]

**Figure S1.** Dissected right knee (salamander 06). A) flexor view; B) flexor view joint detail; C) posterior view; D) extensor view; E) extensor view joint detail; F) anterior view. Skin and muscle was removed but the ligaments and joint capsule were left intact for the *ex vivo* experiments. Scale bar = 1 cm.

**Figure S2.** Rigging of a right knee joint (salamander 08). Steps are as follows: 1) proximal and distal bones of the dissected specimen were placed on acrylic plates; 2) proximal bone (femur) was attached to the acrylic plate via wire, which was wrapped and glued to the plate; 3) glue was used to secure the wire fixing the proximal bone, and the distal bone was attached to its plate with wire; 4) the distal bone was fixed to the plate with glue. A) anterior view, with the flexor surface of the knee joint pointing up; B) view of flexor surface of knee joint.

**Figure S3.** Results of the ligament-only *ex vivo* hip joint RoM, obtained from motion capture tests. A) Right hip, anterolateral view; B) Right hip, posterolateral view. C,E,G,I) SFPs from anterolateral view; D,F,H,J) SFPs from posterolateral view. C,D) Salamander 10, trials 1-6. E,F) Salamander 12, trials 1-5. G,H) Salamander 13, trials 1-5. I,J) Salamander 14 left hip, trials 1-5, data from left hip transformed to visualise right hip RoM for consistency with other specimens. Units on axes refer to length of unit vectors of the axes of the acetabular coordinate system.

**Figure S4.** Results of the ligament-only *ex vivo* knee joint RoM, obtained from motion capture tests. A) Right knee, anteroventral view, showing distal femur and proximal tibia/fibula ACSs. The ABAD (Y) axis points up, in a similar arrangement to the setup in the rig. B) Right knee, ventral view, with positive ABAD (Y) axis pointing out of page. C-F) SFPs for ligament-only knee joints. C,D) SFP for salamander 06 right knee, trials 1-6, in C) anteroventral and D) ventral views. E,F) SFP for salamander 08 right knee, trials 1-6, in E) anteroventral and F) ventral views. In these SFPs, points on the sphere show the excursion of the proximal ACS of the tibia/fibula, relative to the distal ACS of the femur. The SFP is a visual representation of the RoM and interaction of degrees of freedom at the joint. Units on axes refer to length of unit vectors of the axes of the distal femur coordinate system.

**Figure S5.** Bone scans of individual pelves (A,C,E,G,I) and femora (B,D,F,H,J) for specimens used in the hip joint experiments. A,B) salamander 10; C,D) salamander 12; E,F) salamander 13; G,H) salamander 14 (left side converted to right side via mirroring and adjusting ACS); I,J) salamander 22 (*in vivo* individual). All bones were aligned in the same orientation based on their anatomical coordinate systems. Pelves in lateral (slightly ventral) view, femora in ventral view. Uneven sections in the model are due to CT scan attenuation due to rigging wires. Scale bars = 5 mm.

**Figure S6.** Bone scans of individual femora (A,C,E, shown in ventral, dorsal, and distal view from left to right) and tibiae/fibulae (B,D,F, shown in ventral, dorsal, and proximal view from left to right) for specimens used in the knee joint experiments. A,B) salamander 06; C,D) salamander 08; E,F) salamander 22 (*in vivo* individual). All bones were aligned in the same orientation based on their anatomical coordinate systems. Uneven sections in the model are due to CT scan attenuation due to rigging wires. Scale bars = 5 mm.
